# Comparative Pharmacokinetics and Bioavailability of Epimedin C in Rat after Intramuscular Administration of Epimedin C, a Combination of Four Flavonoid Glycosides and Purified Herba Epimedii Extract

**DOI:** 10.1155/2016/5093537

**Published:** 2016-08-11

**Authors:** Shunjun Xu, Jiejing Yu, Liu Yang, Yaling Zhu, Shuai Sun, Zhengdi Xu

**Affiliations:** ^1^ImVin Pharmaceutical Co., Ltd., 2 Fangcaodian Road, Guangzhou 510663, China; ^2^Guangdong Provincial Hospital of Chinese Medicine, 111 Dade Road, Guangzhou 510120, China

## Abstract

Chuan-Ke-Zhi (CKZ), a purified Herba Epimedii extract, is a potent Chinese medicine preparation whose main bioactive components are a class of flavonoid glycosides such as epimedins A, B, and C and icariin. And epimedin C is far more abundant than other flavones in this extract. This study aims to investigate the pharmacokinetic and bioavailability of epimedin C and what effects, if any, other ingredients in CKZ have on its pharmacokinetics. Epimedin C, CKZ, and a combination of epimedins A, B, and C and icariin were, respectively, administrated to rats, and then the pharmacokinetic parameters of epimedin C in the three groups were calculated and compared. The result indicated that CL_Z_, MRT_0–∞_, and AUC_0–∞_ of epimedin C were significantly different among the three groups (*P* < 0.05), and compared with the epimedin C group, the absorption of epimedin C significantly increased in the CKZ group. Furthermore, in this study the absolute bioavailability of epimedin C was also investigated by comparing intramuscular and intravenous administration of epimedin C. As a result, epimedin C could be quickly absorbed with extremely high absolute bioavailability after intramuscular administration.

## 1. Introduction

Purified Herba Epimedii extract (Chuan-Ke-Zhi, CKZ), a Chinese medicine preparation, has already attracted more and more attention in recent years for its curative effects on respiratory system diseases, such as bronchial asthma, chronic bronchitis, and chronic obstructive pulmonary emphysema [1–4]. And this purified extract was also reported to be able to regulate human humoral and cellular immunity [5–9]; therefore, it has been gradually extended to the treatment of various cancers and immunological diseases [10–13].

Although CKZ has been clinically applied for many years, there are still some difficulties and challenges on its* in vivo* process and mechanism of action. Nowadays the pharmacokinetic research of traditional Chinese medicine (TCM) are usually carried out in accordance with the research method of western medicine; however, not like western medicine TCM always contains numerous compounds, which generally display different pharmacokinetic behaviour and may interact with each other in organism. Therefore in order to evaluate the rationality and compatibility of Chinese herbal compound formula, it is obviously important and necessary to investigate the pharmacokinetics of marker component as well as the possible interaction of other ingredients contained in TCM.

**Figure 1:**
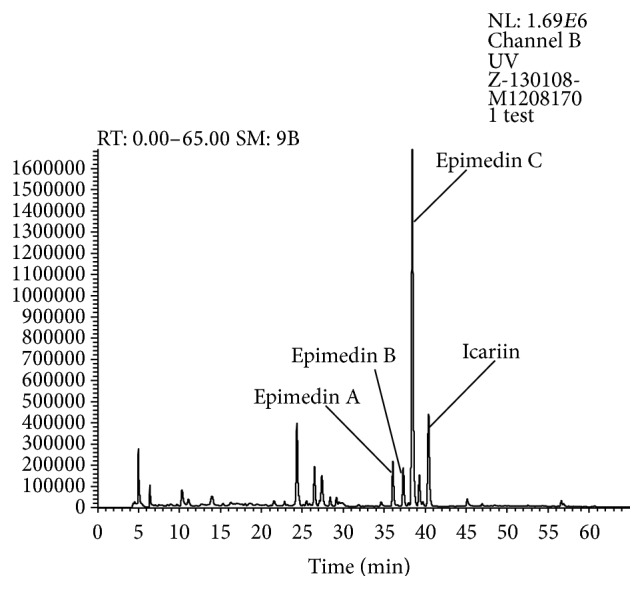
HPLC-DAD fingerprinting of purified Herba Epimedii extract (CKZ) at 270 nm.

According to the existing phytochemical and pharmacological studies [14–20], Herba Epimedii contains a large number of flavonoid glycosides. Most of them are characterized by an isopentenyl group substitution at the C_8_ position of flavonoid skeleton. These 8-isopentenyl flavonoid glycosides, of which epimedins A, B, and C and icariin are the principal and representative, are thought to be main bioactive components of Herba Epimedii and its extract preparation. As a purified extract, CKZ contains more abundant 8-isopentenyl flavonoid glycosides than this crude drug, and epimedin C is the most dominant compound, which is visually exhibited in Figure 1 [21]. In the present study, therefore, the pharmacokinetic research of CKZ firstly focused on the pharmacokinetic behaviour of epimedin C in rat, and then on the base of established and validated quantitative method for epimedin C in rat plasma, we compared the pharmacokinetic differences of epimedin C after intramuscular administration of individual epimedin C, combination solution of epimedins A, B, and C and icariin, and CKZ to rats. Finally, considering the poor oral absorption of epimedin C [22, 23], the absolute bioavailability of epimedin C administrated intramuscularly was investigated for the first time in this study. Overall, our study could facilitate the understanding of absorption mechanism of flavonoid glycosides in herbal formulations and provide reference for the clinical application of this purified Herba Epimedii extract.

## 2. Experimental

### 2.1. Chemicals and Reagents

**Figure 2:**
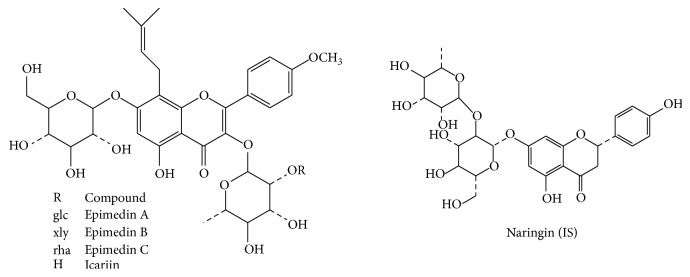
Chemical structure of epimedin A, epimedin B, epimedin C, icariin, and naringin (IS).

Epimedin A (purity 98%) and epimedin B (purity 98%) were purchased from Shanghai Ronghe Pharmaceutical Technology Development Co., Ltd. (Shanghai, China). Epimedin C (purity 98%), icariin (purity 98%), and naringin (purity 98%) used as internal standard (IS) were provided by the National Institute for the Control of Pharmaceutical and Biological Products (Beijing, China). The chemical structures of 4 flavonoid glycosides and IS are shown in Figure 2. CKZ was provided by Guangzhou ImVin Pharmaceutical Co., Ltd. (Lot number 11072502, Guangzhou, China), and the concentration of epimedins A, B, and C and icariin was determined to be 81.38, 68.49, 667.5, and 132.5 *μ*g/mL, respectively. HPLC-grade methanol and acetonitrile were obtained from Fisher Company (Fisher Scientific, Fairlawn, NJ). HPLC-grade formic acid was purchased from Tedia Company, Inc. (Fairfield, OH, USA). The deionized water was prepared by a Millipore purification system (Millipore, Milford, MA, USA) and filtered with 0.2 *μ*m membranes.

### 2.2. Instrumentation and Conditions

HPLC was performed on an Agilent Eclipse XDB-C_18_ (150 × 2.1 mm i.d. 5 *μ*m) column using an Agilent 1100 series HPLC system (Agilent Technologies, Inc., USA) equipped with a G1311A binary pump, a G1379A vacuum degasser unit and a G1313A autosampler, and a G1316A SL thermostated column compartment. The temperature of column chamber was set at 40°C. The mobile phase consisted of acetonitrile and 0.1% formic acid (35 : 65, v/v). An isocratic elution mode was adopted with a flow rate of 220 *μ*L/min. The HPLC system was coupled to a triple-quadruple tandem mass spectrometer (API-3000 Pulsar, Applied Biosystems, Foster City, USA) equipped with an electrospray ionization (ESI) source. Analyst 1.4 Workstation (Applied Biosystems, Foster City, USA) was used for the control of equipment, data acquisition, and analysis.

For the optimization of MS/MS parameters, the standard solutions of epimedin C and IS prepared with 50% acetonitrile were infused at a flow rate of 20 *μ*L/min using a syringe pump (Harvard Apparatus, Holliston, MA, USA). Finally, the instrument was operated with the ionspray voltage, deflector voltage (DF) and multiplier voltage (CEM) at −5000, −200, and 2200 V, respectively. Nitrogen was used as nebulizer gas (8 L/min), as auxiliary gas (7.5 L/min at 350°C), and as curtain gas (8 L/min). Multiple reaction monitoring (MRM) was employed for data acquisition. The optimized MRM fragmentation transitions were* m/z *867.3 → 659.3 for epimedin C and* m/z *579.2 → 271.1 for IS. The dwell time for each transition was 200 ms.

### 2.3. Animal

In this study, the experimentation on rats obtained the approval from an independent ethics committee at Guangdong Provincial Hospital of Chinese Medicine (approval number 2012038). The experiment was performed at an SPF level laboratory, authorized by Guangdong Provincial Government. Sprague-Dawley rats (300 ± 20 g) were purchased from Guangdong Medicinal Laboratory Animal Center. The animals fasted for 12 h with free access to water prior to administration.

### 2.4. Drug Administration and Blood Sampling

Twenty-four Sprague-Dawley rats randomly divided into four groups. The four groups were administrated as follows: two groups dosed with epimedin C aqueous solution by intramuscular and intravenous administration, respectively, one group dosed with CKZ by intramuscular administration, and one group intramuscularly administrated with the combination solution of epimedins A, B, and C and icariin whose contents are the same as those in CKZ. The four aqueous solutions were administered to rat at the same epimedin C dosage (approximately 0.33 mg/kg).

About 350 *μ*L of blood sample was collected from the suborbital vein into heparinized tubes before drug administration (0 h) and at 3, 6, 10, 15, 25, 35, and 45 min and 1, 2, 3, 4, 6, and 8 h after intramuscular and intravenous administration. And then each blood sample was centrifuged at 3000 rcf for 15 min at 4°C, and the plasma was transferred into 1.5 mL centrifuge tube and stored at −80°C until analysis. During routine analysis, each analytical run included blank rat plasma, a set of calibration samples, and a set of QC samples.

### 2.5. Plasma Sample Preparation

A simple solid-phase extraction (SPE) method was used for extracting epimedin C from rat plasma. The Agilent ODS-C_18_ SPE cartridge was preconditioned with 1 mL methanol followed with 1 mL water. A 100 *μ*L aliquot of plasma sample was spiked with 10 *μ*L of IS working solution (1 *μ*g/mL). The combination was vortexed for 60 s and then added to a pretreated cartridge. After the sample was drawn through the SPE bed, the cartridge was washed with 1.0 mL water, followed by vacuumizing for 1 min to remove the aqueous effluent. The cartridge was then eluted with 1.0 mL methanol. The methanol eluate was transferred to a clean test tube and evaporated to dryness under a gentle stream of nitrogen at 40°C. The residue was redissolved in 150 *μ*L of mobile phase (35% acetonitrile containing 0.1% formic acid) and centrifuged by a microspin filter tube (0.22 *μ*m nylon, Alltech) at 10,800 ×g for 1 min. A 10 *μ*L aliquot of the solution was injected into LC-MS/MS for analysis.

### 2.6. Method Validation

Blank rat plasma samples were spiked with epimedin C standard solutions to determine the final concentration ranges (1, 3, 10, 30, 100, 300, and 1000 ng/mL). Linearity was assessed by assaying calibration curves on three consecutive days. And the curves were fitted by a weighted (1/*x* ^2^) least-squares linear regression method through the measurement of the peak-area ratio of epimedin C to IS. The acceptance criterion for a calibration curve was a correlation coefficient (*r*) of 0.99 or better, and each back-calculated standard concentration must be within 15% deviation from the nominal value except at the lower limit of quantitation (LLOQ), for which the maximum acceptable deviation was set at 20%. The LLOQ was defined as the lowest concentration on the standard curve at which the standard deviation was within 20% and accuracy was within 100 ± 20%, and it was established using five samples independent of standards. The analyte response at this concentration level should be >5 times the baseline noise.

To evaluate the accuracy and precision of established method, QC samples at four concentration levels (3, 30, 300, and 800 ng/mL) were analyzed in five replicates on three validation days. The assay accuracy was expressed as (observed concentration/nominal concentration) × 100%. Intra- and interday precision were expressed as relative standard deviation (RSD). The accuracy was required to be within 85–115%, and the intra- and interday precision are not to exceed 15%.

To investigate the effect of the sample preparation, the extraction recovery and matrix effect were determined by comparing the peak areas between three types of samples: (1) plasma spiked with known amount of epimedin C before sample preparation; (2) plasma spiked with known amount of epimedin C after sample preparation; (3) the standard solution of epimedin C. The difference between peak areas of sample 2 and sample 3 reflects the extent of ion suppression. The difference between peak areas of sample 1 and sample 2 reflects the recovery. The accuracy (trueness) of the method was calculated by comparing theoretical and experimentally measured analyte levels.

The stability of epimedin C in rat plasma was evaluated using 20 QC samples (five samples at each concentration). The stability was tested under the following conditions: (1) freeze-thaw stability of epimedin C in rat plasma through three freeze-thaw cycles; (2) short-term stability of epimedin C in rat plasma at room temperature for 6 h; (3) long-term stability of epimedin C in rat plasma stored at −80°C for 30 days; (4) postpreparative stability of epimedin C in rat plasma stored in the autosampler at 25°C for 24 h.

### 2.7. Pharmacokinetic Analysis

The validated method was applied to the pharmacokinetic and bioavailability studies of epimedin C in rats. The pharmacokinetic parameters were analyzed by noncompartmental methods. The maximum plasma concentration (*C*_max_) and time to *C*_max_ (*T*_max_) were obtained directly from the concentration-time curves. The area under the concentration-time curve (AUC_0–*∞*_) was calculated using linear trapezoidal rule. The mean residence time (MRT) was calculated as AUMC_0–*∞*_/AUC_0–*∞*_, where AUMC_0–*∞*_ represents the area under the first moment plasma concentration-time curve. All data were analyzed by DAS Software (ver. 2.0, China State Drug Administration).

## 3. Results and Discussion

### 3.1. Method Development

**Figure 3:**
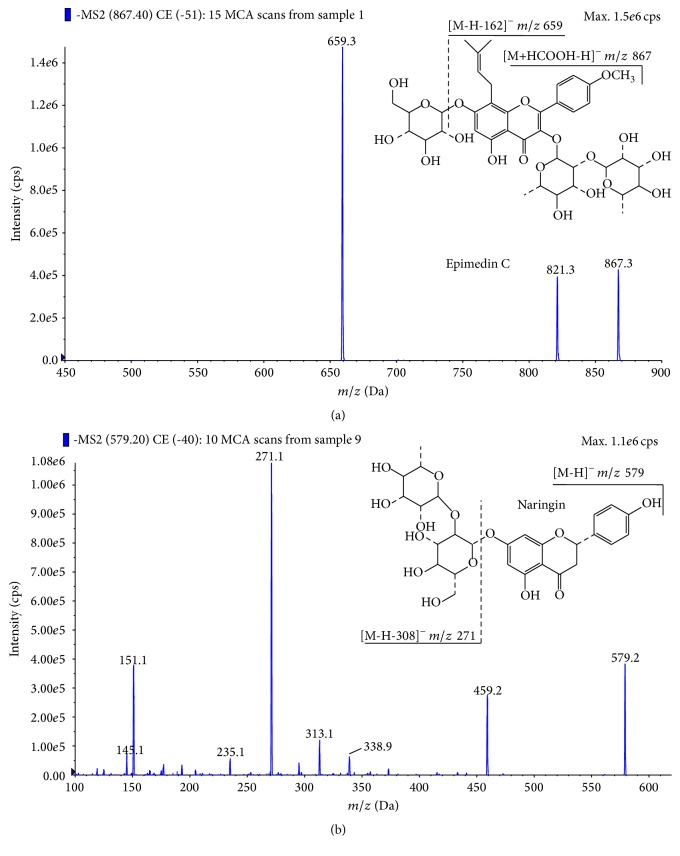
MS/MS spectra of (a) epimedin C (*m/z *867.3 → 659.3) and (b) naringin (IS,* m/z *579.2 → 271.1).

The mass spectrometric behaviour of epimedin C and IS was studied using both positive and negative ion ESI. It was found that epimedin C and IS had good responses in negative ion detection mode with a low background noise level. Detection was finally operated in negative ion mode in this study. The MS/MS spectra of epimedin C and IS were shown in Figure 3.

Protein precipitation and SPE were compared for sample preparation. The former was discarded because of its high noise level and the interference of endogenous substances. The latter offered a very clean sample which made the quantitative method robust and scalable. Consequently, the SPE method was used for sample preparation and further optimized by testing and comparing the influence of different solid-phase extraction cartridges (C_8_, C_18_, and HLB) on the recovery of analyte and IS. Agilent C_18_ SPE cartridge was the best owing to its low background noise, ease of sample preparation, and relatively high extraction recovery. The optimal conditions were presented above.

**Figure 4:**
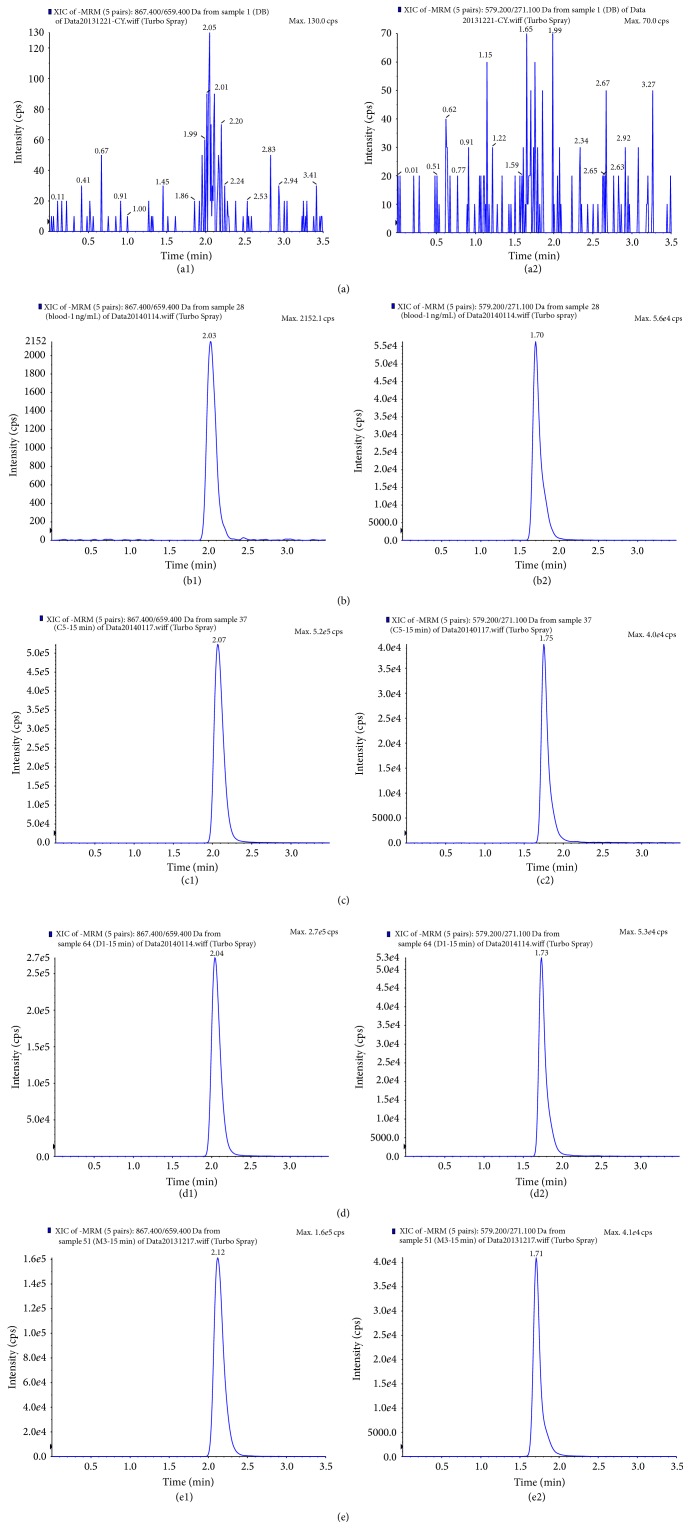
Typical MRM chromatograms of epimedin C (1) and IS (2). (a) Blank rat plasma; (b) blank plasma sample spiked with epimedin C at LLOQ and IS (66.7 ng/mL); (c) rat plasma sample at 15 min after intramuscular injection of epimedin C; (d) rat plasma sample at 15 min after intramuscular administration of combination solution of four flavonoid glycosides; (e) rat plasma sample at 15 min after intramuscular administration of CKZ.

In order to improve the peak shape and enhance the signal response of analytes, and to reduce the run time, different analytical columns and mobile phase compositions were tried to achieve good resolution and symmetric peak shapes for epimedin C and IS. By comparison with Shiseido capcell pak C_18_ (150 × 2.0 mm i.d. 5 *μ*m) column, Agilent XDB C_18_ (150 × 2.1 mm i.d. 5 *μ*m) column could obtain better chromatographic behaviour and higher signal response for the analytes. At last, the Agilent XDB C_18_ (150 × 2.1 mm i.d. 5 *μ*m) column was selected for analysis. The modifier of mobile phase of acetonitrile-water binary solvent system was screened from ammonium acetate, acetate acid, and formic acid. As a result, the mobile phase consisting of acetonitrile-water containing 0.1% formic acid with an isocratic elution could improve the symmetry of peak shape and enhance the signal response. The flow rate was also optimized and finally set at 0.22 mL/min. Under the above-mentioned HPLC conditions, the retention time of epimedin C and IS was within 3 min, and no interfering substance was detected at the retention time of analytes in blank rat plasma samples (shown in Figure 4). The overall chromatographic run time was performed within 3.5 min.

### 3.2. Method Validation

The specificity of the analytical method was evaluated by analyzing individual blank plasma samples from six different sources. All samples were found to have no interference with endogenous substances and IS at the respective retention position of epimedin C. Typical MRM chromatograms of blank plasma spiked with IS, plasma spiked with epimedin C, and the rat plasma sample after intramuscular administration of epimedin C, flavone combination, and CKZ were shown in Figure 4. The regression equation, linear range, and correlation coefficient of epimedin C were *Y* = 0.00461*X* + 0.002 (1.00–1000 ng/mL) and *R* ^2^ = 0.9981. The LLOD with a signal-to-noise (*S*/*N*) ratio of >5 of epimedin C was 0.04 ng/mL, while the LLOQ with an *S*/*N* ratio of >10 was 0.05 ng/mL, which was sensitive enough for pharmacokinetic study of epimedin C in rat plasma. Intra- and interday precision and accuracy were calculated by analysis of variances, based on replicate analyses (three days, four concentrations, each *n* = 5) of QC samples. The data was listed in Table 1. The mean extraction recovery rates of epimedin C from rat plasma were 86.6 ± 3.1, 80.7 ± 4.3, 80.1 ± 2.6, and 78.4 ± 1.1 at low, medium, and high concentrations, and the matrix effects were 90.71 ± 5.3, 95.95 ± 2.49, 94.57 ± 0.82, and 100.9 ± 2.21, respectively. The result of stability experiment showed that epimedin C was stable in autosampler (24 h) at 25°C, bench-top (6 h) at room temperature, and under repeated three freeze/thaw cycles and frozen condition at −80°C within 30 days (Table 2), as RSDs were within ±15% for both the low and high concentrations. The method showed good precision, repeatability, and stability and was suitable for determination of epimedin C in biological samples.

### 3.3. Pharmacokinetic Study

**Figure 5:**
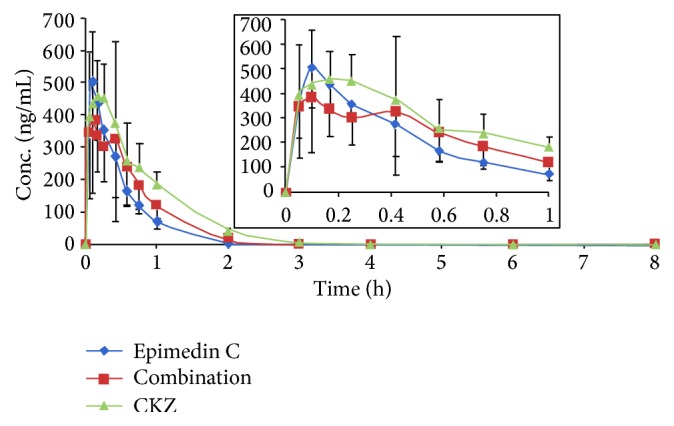
Mean plasma concentration-time profiles of epimedin C after intramuscular administration of epimedin C, combination of four flavonoid glycosides, and CKZ at the epimedin C dose of 0.33 mg/kg (*n* = 6).

After administration of epimedin C, CKZ, and combination of epimedins A, B, and C and icariin, the plasma epimedin C concentrations were successfully determined by LC-MS/MS method described above. The mean plasma concentration-time profiles of epimedin C were illustrated in Figure 5. The major pharmacokinetic parameters and absolute bioavailability were shown in Table 3. The result indicated that epimedin C exhibited rapid absorption with the plasma peak concentrations occurring at around 10 min and were quickly eliminated thereafter. In addition, the plasma concentration-time curves of epimedin C after intramuscular administration showed a second peak or shoulder in most rats. The phenomenon might relate with enterohepatic recycling or release of tissue-bound drug.

As shown in Table 3, the data indicated that many pharmacokinetic parameters were significantly different among three intramuscular administration groups. Values of the area under the plasma concentration versus the time curve (AUC_0–*∞*_) were 271.03 ± 44.39, 311.41 ± 128.11, and 448.45 ± 137.74 for intramuscular administration of epimedin C, combination of four flavones, and CKZ, respectively. Compared with administration of individual epimedin C, AUC of epimedin C in CKZ group was statistically different (*P* < 0.05), while after administration of combination of four flavones, the AUC value was also higher than that of individual epimedin C but the difference was not significant. The MRT_0–*∞*_ and* T*_1/2_ values of epimedin C after administration of CKZ and combination of four flavones were longer than corresponding values after administration of epimedin C, and the MRT_0–*∞*_ value was significantly different (*P* < 0.01) among three groups. Drug clearance (CL_Z_) is defined as the volume of plasma that would contain the amount of drug excreted per minute. In the present study, drug clearance (CL_Z_) of epimedin C was 1.26 ± 0.21, 1.20 ± 0.41, and 0.79 ± 0.19 for epimedin C, combination, and CKZ group, respectively. The data demonstrated that the systemic clearance of epimedin C is significantly different (*P* < 0.01) between epimedin C group and CKZ group. In summary, longer MRT_0–*∞*_ and* T*_1/2_, higher AUC, and lower CL_Z_ values of epimedin C in CKZ group than epimedin C group implied that the purified herbal preparation may be superior to single compound in prolonging the duration of action of epimedin C.

The bioavailability of flavonoids has received extensive attention for many years due to its poor oral absorption characteristic. Information on the comparative kinetic and bioavailability studies of flavonoid glycosides from Herba Epimedii is important for understanding the biological effects of the herbal drug and its preparation. It has been reported that the oral bioavailability of individual epimedin C was higher than that of Herba Epimedii extracts [22]. Our results, on the contrary, turned out that, after intramuscular administration of the purified extract to rats, the absorption and bioavailability of epimedin C enhanced significantly in comparison with those of individual epimedin C administered intramuscularly, which suggests other components in CKI could improve the bioavailability of epimedin C. Because of the synergistic effects of other herbs/components, Chinese herbal formula is usually more potent than individual herb/component, which has been confirmed by more and more researches on Chinese medicine formula such as Huangqin decoction [24–27]. The purified herbal preparation might be more effective than individual epimedin C via intramuscular administration in clinical application. Moreover, the above-mentioned report also demonstrated that epimedin C is poorly absorbed following oral administration of both individual compound and herbal extract and the absolute bioavailability of epimedin C is not more than 0.58% in rat. In the present study, we observed, compared to the oral administration, that epimedin C was quickly absorbed with an extremely high bioavailability after intramuscular administration; the absolute bioavailability of this flavonoid glycoside was about 100%. Clearly, an injectable form of epimedin C may be more effective and reasonable for the clinical application of the purified Herba Epimedii extract preparation.

## 4. Conclusions

Our study was designed to evaluate the pharmacokinetic parameters of epimedin C after a single dose of epimedin C, combination of four flavonoid glycosides, and CKZ administered, respectively, to rats via intramuscular and intravenous injection and to compare the relative and absolute bioavailability of epimedin C. Although there have been investigations on the pharmacokinetics of epimedin C administrated orally [22, 23], to the best of our knowledge, this is the first study to reveal the comparative pharmacokinetic and absolute bioavailability of epimedin C administrated intramuscularly. The results showed that among three intramuscular administration groups the differences of pharmacokinetic parameters such as CL_Z_, MRT_0–*∞*_, and AUC_0–*∞*_ were statistically significant (*P* < 0.05). With the obtained pharmacokinetic results, it can be concluded that (1) after intramuscular administration of the purified Herba Epimedii extract to rats, the absorption and bioavailability of epimedin C enhanced significantly in comparison with those of epimedin C administered individually. In brief, other ingredients in the purified extract can affect the pharmacokinetic behaviour of epimedin C in rats; (2) as for epimedin C, intramuscular administration is superior to oral administration due to higher absolute bioavailability; (3) the results presented in this study implicated that the purified herbal preparation might be more effective and safer than individual compound. In conclusion, results from this work could provide some guidance for the clinical applications of this herbal drug extract. And in terms of the bioavailability of epimedin C, intramuscular administration of CKZ should be reasonable and effective in clinical application.

## Figures and Tables

**Table 1:** Intra- and interday precision and accuracy of epimedin C in rat plasma (*n* = 5).

Concentration (ng/mL)	Intraday (*n* = 5)		Interday (*n* = 3)	
	Accuracy (%)	RSD (%)	Accuracy (%)	RSD (%)
3	105.4	4.7	105.0	3.7
30	106.2	3.2	99.8	6.7
300	107.5	4.1	96.5	10.2
800	98.4	2.2	92.7	5.8

**Table 2:** Stability data of epimedin C in rat plasma (*n* = 5).

Concentration (ng/mL)	Accuracy (%) (mean ± SD)			
	Short-term Mean ± SD	Postterm Mean ± SD	Long-term Mean ± SD	Freeze-thaw Mean ± SD
3	99.5 ± 12.0	101.8 ± 8.6	93.9 ± 6.0	100.5 ± 5.3
30	99.8 ± 10.7	100.7 ± 6.5	94.7 ± 1.7	102.7 ± 4.0
300	90.9 ± 3.9	91.2 ± 2.3	102.5 ± 0.8	100.7 ± 2.3
800	88.4 ± 1.3	88.4 ± 1.8	105.8 ± 2.4	91.3 ± 2.8

**Table 3:** The pharmacokinetic parameters of epimedin C after intramuscular administration of epimedin C, combination of four flavonoid glycosides, and CKZ at the epimedin C dose of 0.33 mg/kg (*n* = 6).

Parameters	Intramuscular administration			Intravenous administration	Bioavailability
	CKZ	Combination	Epimedin C	Epimedin C	*F* (%)
*T*_1/2_ (h)	0.47 ± 0.08	0.39 ± 0.05	0.33 ± 0.14	0.32 ± 0.07	
*C*_max_ (*µ*g/L)	468.13 ± 135.83	419.83 ± 225.30	515.48 ± 162.72	696.81 ± 83.69	
*V*_Z/F_ (L/kg)	0.53 ± 0.13	0.65 ± 0.23	0.59 ± 0.25	0.62 ± 0.12	
CL_Z_ (L/h/kg)	0.79 ± 0.19^*∗∗*^	1.15 ± 0.39	1.26 ± 0.21	1.35 ± 0.16	
MRT_0–*∞*_ (h)	0.77 ± 0.15^*∗∗*^	0.69 ± 0.06^*∗∗*^	0.52 ± 0.12	0.25 ± 0.04	
AUC_0–*∞*_ (*µ*g/L·h)	448.45 ± 137.74^*∗*^	326.59 ± 139.081	271.03 ± 44.39	246.45 ± 27.49	109.97

Data versus those of intramuscular administration of epimedin C, ^*∗*^ *P* < 0.05; ^*∗∗*^ *P* < 0.01.

*F* = [AUC_0–*∞*_^intramuscular^]/[AUC_0–*∞*_^intravenous^] × 100%.
